# Supplementary figures and images for: Mechanical feedback and robustness of apical constrictions in Drosophila embryo ventral furrow formation
Source: PLoS Comput Biol. 2021 Jul 6;17(7):e1009173. doi: 10.1371/journal.pcbi.1009173 (PMC8284804; doi:10.1371/journal.pcbi.1009173)

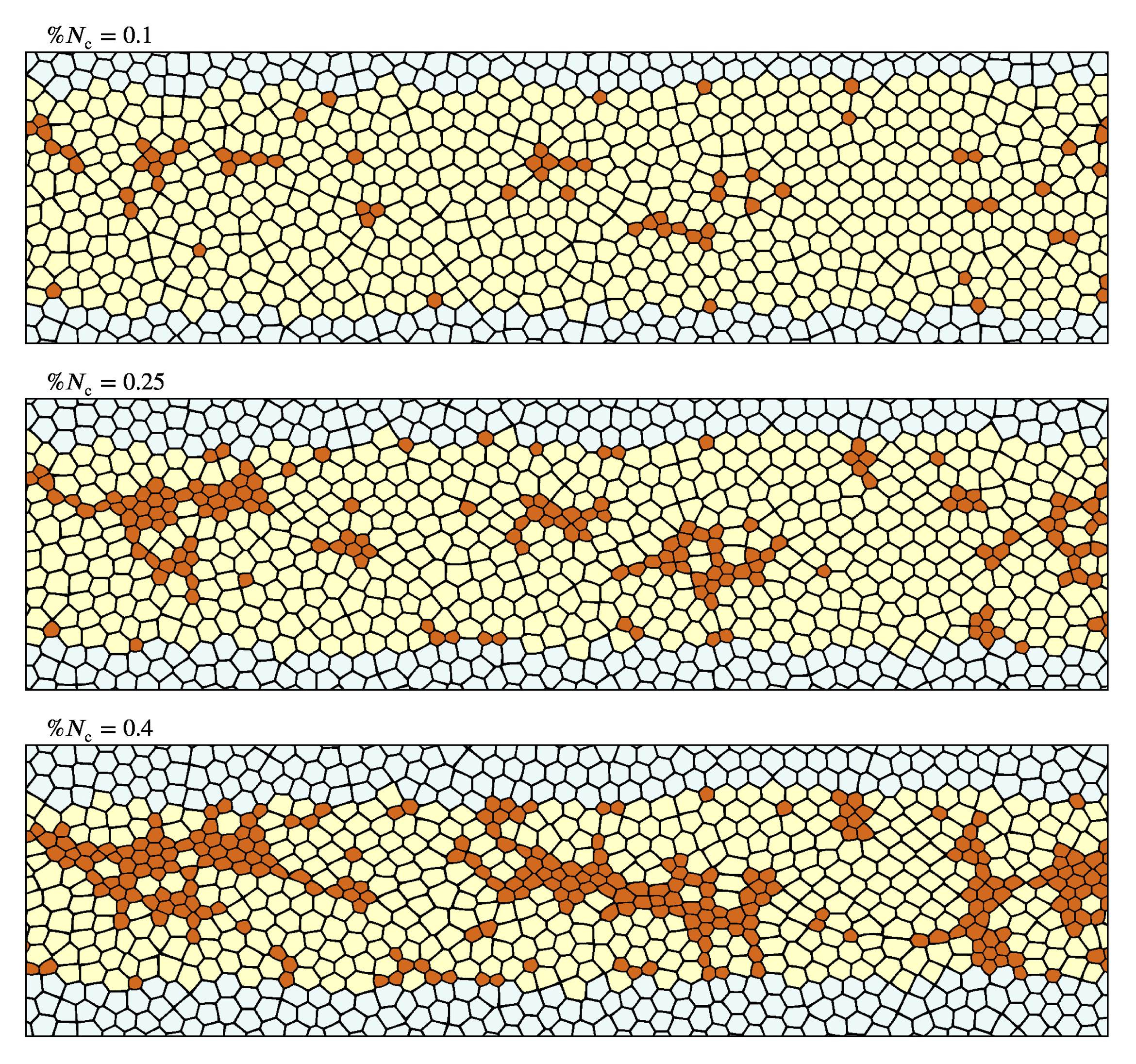

Supplement: S1 Fig — A developing constriction pattern is shown for a model system in which unconstricted active cells with nc constricted neighbors have their constriction probability increased by a factor βcn = 1 + 3.33nc. Unlike tensile feedback, neighbor-driven constriction enhancement does not lead to formation of a connected network of constriction chains. (TIFF) [file pcbi.1009173.s001.tiff]

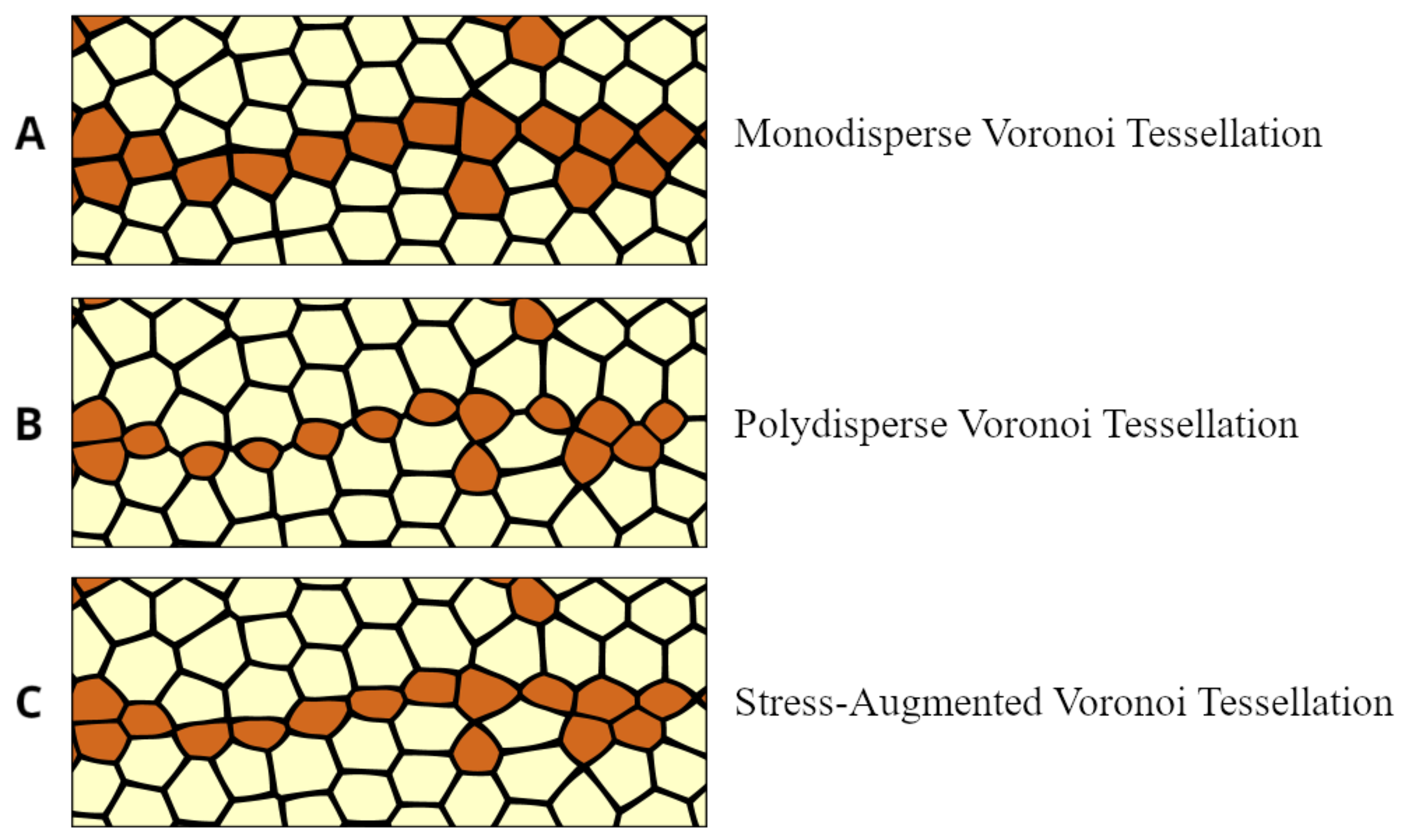

Supplement: S2 Fig — (A) The standard monodisperse tessellation assumes that all cells are size-weighted equally and draws membranes exactly halfway between cell centers, as described by the shape tensor Di = I in Eq 11. (B) The polydisperse tessellation moves the membranes closer to smaller cells and further from larger cells based on the ratio of their sizes (Di = di I). (C) The stress-augmented tessellation weights membrane placement between two cells based on the mechanical stress that each cell experiences [Di=di(I+s0-1Si), where S is the virial stress, as described in more detail in the main text]. The stress-augmented Voronoi algorithm renders the most realistic representation of a confluent cellular medium with chains of constricted cells (brown). (TIFF) [file pcbi.1009173.s002.tiff]

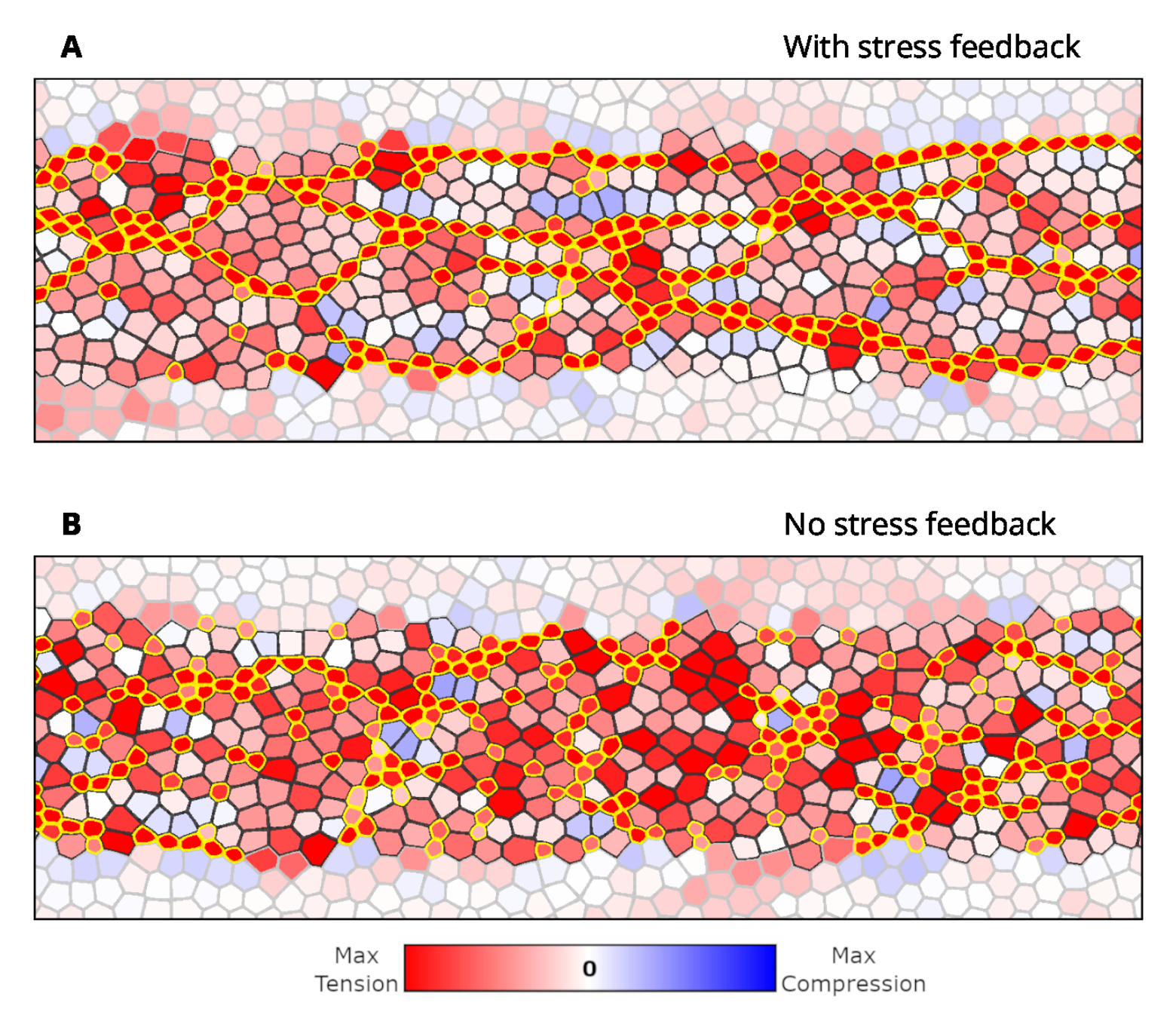

Supplement: S3 Fig — In the system with tensile feedback (top) tensile stress is supported by chains of constricted cells, while unconstricted cells are subject to relatively small tensile forces. In the random uncorrelated system (bottom) there are large groups of unconstricted cells that bear strong tensile stress. Results are shown for a system with 40% of cells constricted; the color scheme and system parameters per Fig 8. (TIFF) [file pcbi.1009173.s003.tiff]

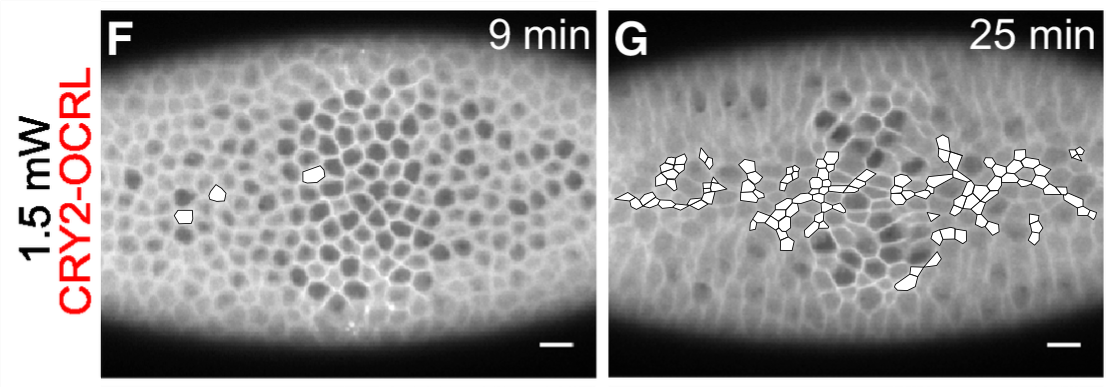

Supplement: S4 Fig — Constricted cells for the 1.5 mW case were manually identified based on overall size relative to other cells in frame and relative visual difference between the major and minor axis of each cell’s individual shape. Scale bars: 10 μm. Figure modified and reprinted from [25] under the article’s CC BY license. (TIFF) [file pcbi.1009173.s004.tiff]

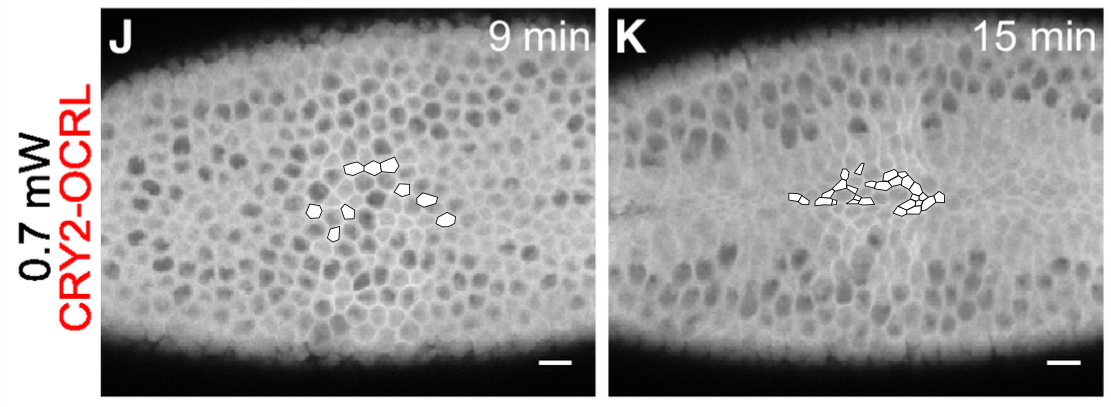

Supplement: S5 Fig — Constricted cells for the 0.7 mW case were manually identified based on overall size relative to other cells in frame and relative visual difference between the major and minor axis of each cell’s individual shape. Only the optogenetically affected area and the area immediately surrounding it were considered due to low contrast of the unaffected areas in (K). As the edge of the forming furrow can be seen in the left side of (K), this low contrast is likely the result of the unaffected areas beginning to invaginate. Scale bars: 10 μm. Figure modified and reprinted from [25] under the article’s CC BY license. (TIFF) [file pcbi.1009173.s005.tiff]

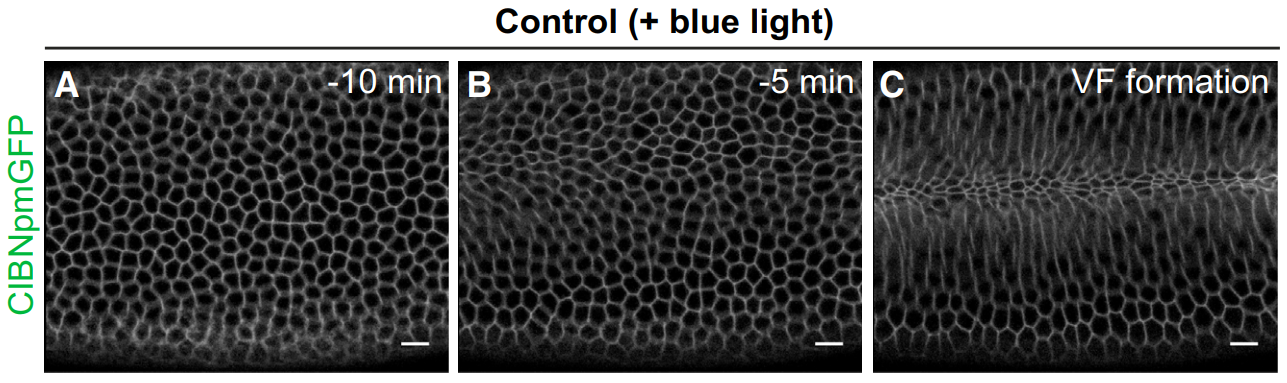

Supplement: S6 Fig — The apical surface of the ventral mesoderm of a wild-type control embryo expressing only CIBN::pmGFP. Frames show the embryo (A) 10 min before, (B) 5 min before, and (C) at the onset of ventral furrow formation. Scale bars: 10 μm. Figure reprinted from [25] under the article’s CC BY license. (TIFF) [file pcbi.1009173.s006.tiff]
