# Supplementary material for: Mechanical feedback and robustness of apical constrictions in Drosophila embryo ventral furrow formation
Source: PLoS Comput Biol. 2021 Jul 6;17(7):e1009173. doi: 10.1371/journal.pcbi.1009173 (PMC8284804; doi:10.1371/journal.pcbi.1009173)
Supplement: S1 Appendix — (PDF) [file pcbi.1009173.s007.pdf]

Orientation of constricted-cell clusters.

Here we discuss the orientation of constricted-cell clusters with respect to the anteroposterior axis of the embryo. In experimental images of the *Drosophila* embryos the cluster orientation is determined from the direction of the line that connects two most distant pixels in the set of cells that form a given cluster. In numerical simulations, the cluster orientation is determined using two the most distant points in the stress-based Voronoi representation of the cells forming the cluster.

The overall cluster orientation is described using the orientational order parameter

$$\psi_2 = \langle \cos 2\phi \rangle_{\mathrm{w}}, \tag{A1}$$

where ϕ is the angle between the anteroposterior axis and the cluster axis. The average $\langle \ldots \rangle_{\mathrm{w}}$ over all clusters ($n_{\mathrm{c}} \geq 2$) identified in the video images or simulation frames corresponding to a given bin of the fraction $\%N_{\mathrm{c}}$ of active constricted cells is weighted with the cluster size n_{c},

$$\langle X \rangle_{\mathrm{w}} = \frac{\displaystyle\sum_{i \in \mathrm{clusters}} n_{\mathrm{c}}(i) X(i)}{\displaystyle\sum_{i \in \mathrm{clusters}} n_{\mathrm{c}}(i)}, \tag{A2}$$

where i is the cluster number, $n_{\mathrm{c}}(i)$ is the particle number in the cluster i, and $X(i)$ is the averaged quantity.

According to the definition Eq A1 of the order parameter ψ_2, clusters completely aligned with and those normal to the anteroposterior axis yield $\psi_2 = 1$, and $\psi_2 = -1$, respectively. A system with a completely random, unbiased angular cluster distribution is characterized by $\psi_2 = 0$.

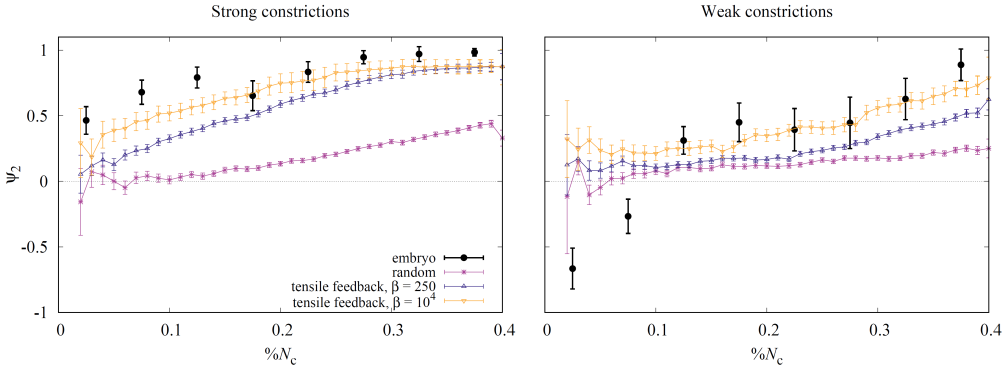

Fig A1. Orientation of constricted-cell clusters in the AGF model and *in vivo*. The orientation parameter ψ_2, Eq. (A1), is plotted vs the fraction of active constricted cells $\%N_{\mathrm{c}}$. Simulation results are shown for the random, stress-insensitive system and for two systems with tensile stress feedback (the stress coupling parameter β as labeled). The results are presented for strong constrictions (left) and weak constrictions (right). Strong and weak constrictions in embryos are identified using constriction thresholds $r_{\mathrm{c}} = 0.65$ and $r_{\mathrm{c}} = 0.85$, respectively. The corresponding constriction factors in simulations are $f_{\mathrm{c}} = 0.6$ and 0.85. These parameters are the same as those used in Fig. 9. Embryos ($n = 5$); simulations ($n = 40$). Error bars: SE.

The angular order parameter ψ_2 evaluated for constricted-cell clusters in *Drosophila* embryos is presented in Fig A1 along with the simulation results for the tensile-stress sensitive and stress-insensitive systems. The cluster orientations are presented both for strong constrictions ($r_c = 0.65$) and weak constrictions ($r_c = 0.85$).

Clusters of strongly constricted cells *in vivo* are strongly aligned with the anteroposterior axis (Fig A1), consistent with the individual video images shown in Fig. 2 (in the main text). The alignment is much weaker for weakly constricted cells. We note that at the initial stage of the constriction process, weakly constricted cells tend to form clusters oriented in the dorsoventral direction, as manifested by the negative values of the order parameter ψ_2. This behavior, also seen in the top panels of Fig. 3 (main text), may indicate that the tissue in the active ventral domain is initially prestressed in the dorsoventral direction, and the anteroposterior tension develops as the constrictions progress.

The model with tensile feedback captures the most important features of the cluster orientation: clusters of strongly constricted cells are highly aligned with the anteroposterior axis; for weakly constricted cells the alignment is significantly less pronounced because of the reduced stresses. The simulations with the stress-coupling parameter $\beta = 250$ [i.e., the same value as the one used in Fig. 9 (main text) to describe the cluster size data] somewhat underestimate the alignment, especially for small fractions of constricted active cells in the strong-constriction case. A closer agreement is obtained using a larger value of $\beta = 10,000$.

The need to use a larger value of β to obtain a better fit between experimental and numerical orientation data may stem from the fact that in our model the constrictions are instantaneous, in contrast to the gradual pulsatile constriction process *in vivo*. Partially constricted cells that are present in live embryos, but not in our model, produce additional tensile stress that enhances cluster alignment but affects the cluster-size distribution less. We will investigate this mechanism in a future publication. Here, however, we choose to keep our model simple by approximating constrictions as instantaneous and using only a single parameter β to fit our data.
