# Supplementary material for: Mechanical feedback and robustness of apical constrictions in Drosophila embryo ventral furrow formation
Source: PLoS Comput Biol. 2021 Jul 6;17(7):e1009173. doi: 10.1371/journal.pcbi.1009173 (PMC8284804; doi:10.1371/journal.pcbi.1009173)
Supplement: Attachment — Submitted filename: List_of_changes.pdf [file pcbi.1009173.s008.pdf]

List of changes

We have added Author Summary and introduced some minor formatting adjustments.
