# Supplementary material for: Mechanical feedback and robustness of apical constrictions in Drosophila embryo ventral furrow formation
Source: PLoS Comput Biol. 2021 Jul 6;17(7):e1009173. doi: 10.1371/journal.pcbi.1009173 (PMC8284804; doi:10.1371/journal.pcbi.1009173)
Supplement: Attachment — Submitted filename: review_PCOMPBIOL_D_20_01470.pdf [file pcbi.1009173.s009.pdf]

This work by Holcomb and colleagues addresses the formation of the ventral furrow that occurs during fruit fly embryogenesis. The paper is a follow-up of Gao et al. 2016, from the same group. The authors of the cited paper proposed, based on computational experiments, that mechanical stress may have an instructive role in cell constriction and promote the formation of cellular constriction chains (CCCs) that are necessary for successful invagination of the embryo. Holcomb et al propose a quantitative comparison between the predicted dynamics and experimental observations, as well as new predictions on the robustness of the process.

In the first part of the manuscript, they show the progressive formation of CCCs in the fly embryo using in vivo imaging and image analysis. Then they introduce a particle-based cellular model (based on Gao et al., 2016) allowing to model mutual cell repulsion and adhesion, as well as stochastic cell constriction as a response to mechanical stress. They derive a number of very interesting results. First, they propose that cells initiate CCCs according to an initial heterogeneous pattern of stress. Cells that are subject to higher tensile stress are more likely to shrink, which in turns amplify the nonuniformity of the stress field, in a potentially very general feedback loop. The authors also assess the robustness of CCC formation with respect to localised constriction defects, and demonstrate that a stress feedback allows to rescue normal cell constriction in a number of scenarios. This last result is compared to previously-published experimental analyses.

I found this paper very stimulating and interesting in general. The general mechanism proposed and explored by the authors is remarkably parsimonious, and seems in good agreement with experimental observations. I believe it would well fit the scope of PLOS Computational Biology. Nevertheless, I do have some concerns listed below, that would require major revision.

1. My major comment concerns the mathematical details of the modelling framework. I generally think that the modelling choices and the mathematical expressions chosen by the authors would deserve further justification.

   The distinction between Eq. 4a (repulsion) and 4b (attraction) is not clear to me, and it seems like a rather ad hoc choice. In particular, shouldn't we expect that $V_{\mathrm{r}}(r_{ij})$ will be effectively zero if cells are not adjacent? In this case, combining the two energies into a single one:

   $$V_{ij} = \frac{\epsilon}{2}\left(1 - \frac{r_{ij}}{d_{ij}}\right)^2$$

   would probably be more straightforward. Also, why should adhesion only exist between adjacent cells and not repulsion? Why is the threshold chosen to be the mean diameter?

   As for the stress-based feedback: Why defining an additional triggering stress σ_i instead of taking the trace of the virial stress $\mathbf{S}_i$ for instance?

2. Eq. 9: Will $P_i(s_i)$ be always be ≤ 1?

3. The manuscript does not provide sufficient details on the choice of the simulation parameters or the numerical resolution. This is a limitation in so far as the authors claim to have a quantitative approach. For example, L197: I am surprised by the precision given for parameter $s_0 = 2.1$. Does it have a strong importance on the result?

   In particular Fig. 9 presents quantitative results that show a good agreement between simulation and experiment. However, this result is predicated upon many parameters that are not clearly discussed.

   This is a major limitation that I would like the authors do address.

4. Please provide detailed information on the computational framework. Is the code available somewhere?

5. L723: The rationale behind the choice a periodic boundary condition for the x-axis is not clear to me. Why not simply fixing the x-position of the boundaries? What kind of implementation difficulty would this bring?

6. L116: this statement is a bit weak. Constriction effects could in principle be incorporated into a vertex model, e.g. via an energy penalty on cell areas. I would suggest the authors to elaborate on this point. Also, further details on existing particle-based models would be useful.

7. Eq. 11, a reference on the notion of "virial stress" would be useful.

8. Paragraph *Optogenetic experiments provide evidence of robustness of apical constrictions in live embryos.* The authors make several observations that should be better argued. I surely appreciate that quantification may be difficult. However, statements like "The lowest level of the optogenetic activation [...] reveal constriction chains crossing the affected region of reduced cell contractility in the middle part of the frame" or "the degree of cell contractility reduction is commensurate with the beam power" are not self-evident to me, based on the image provided.

9. Fig 13. Would a control case be available?

10. L571–578: I do not understand this paragraph.

11. An element of discussion: could pulsatile constriction be a way to channel the formation of CCCs and improve the robustness of the process (by introducing some form of 'thermal' noise)?

12. L186: typo "the the".

13. Fig. 1: Note that the bar-circle representation does not make it possible to really visualise the cell surface areas, which anyway do not provide much information I think.

14. L489: Does ν_{avg} actually refer to $\%N_c$?
