# Supplementary material for: Mechanical feedback and robustness of apical constrictions in Drosophila embryo ventral furrow formation
Source: PLoS Comput Biol. 2021 Jul 6;17(7):e1009173. doi: 10.1371/journal.pcbi.1009173 (PMC8284804; doi:10.1371/journal.pcbi.1009173)
Supplement: Attachment — Submitted filename: Responses_1-4.pdf [file pcbi.1009173.s010.pdf]

Response to Reviewer 1

Response to general remarks

Reviewer's Comment: *In this paper, Holcomb et al investigate processes preceding ventral furrow formation in Drosophila, where cells constrict apically before the furrow starts forming. Specifically, the authors consider the spatial coordination of constricting cells. The authors have previously observed that constricting cells in this system form long, connected chains that span in the antero-posterior direction of the embryo. How this spatial alignment occurs mechanistically has not been clear. Here, the authors combine computational modeling and image analysis to show that mechanical feedback can explain the spatial alignment of constricting cells that has been observed experimentally.*

Intriguingly the model captures many qualitative features of these chains. For example, biologically chains of constricting cells grow 'towards each other', which in the model is explained nicely through the mechanical feedback, and the model can quantitatively fit distributions of chain lengths over time. This new explanation of spatial coordination is supplemented with an intriguing analysis of the model, which illustrates that such mechanical feedback can support developmental robustness. In a system where cells constrict due to mechanical feedback, constriction chains can 'invade' or surround regions in which contractility is reduced, leading to a larger fraction of constricting cells than in a scenario where mechanical feedback is absent. These latter results tie in nicely with previously published data from optogenetic experimental perturbations of contractility, and the authors discuss the similarities between their model and these existing data.

Our Reply:
We appreciate the Reviewer's insightful comments and the summary of our key results.

Reviewer's Comment: *I really enjoyed reading this thoroughly conducted and extensive paper. The paper informs multiple topics that are currently important in developmental biology research, namely the investigation of spatial cross-talk between cells on short lengthscales, the role and control of developmental mechanics, as well as the emergence of developmental robustness. This is a timely paper that I believe will make a strong contribution to PLOS Computational Biology.*

I only have a few minor comments that I hope the authors can clarify before publication.

Our Reply:
We thank the Reviewer for his/her positive remarks and for the comments that helped us make our paper clearer.

Response to comments and questions

1. Reviewer's Comment: *How is the model parameterised? Specifically, which model parameters where fitted in figure 9? What were the values of the remaining model parameters and how were these identified?*

   Our Reply:
   Apart from the geometrical parameters such as the number of cells in the simulation domain, the initial size ratio of the cells, and the width of the active region, our model has only three dimensionless parameters: the parameter p in the definition of the feedback parameter, Eq. (6); the constriction factor f_c by which the radius of the interaction potential is reduced upon constriction, Eq. (3); and the coupling strength parameter β, which determines the feedback strength.

The geometrical parameters and the parameter $p = 3$ were kept constant in all our simulations and were not used for fitting. The constriction factor f_c values are defined by $f_c \approx r_c$, where r_c is the cutoff used to identify constricted cells *in vivo* for the two constriction strengths considered. A single fitting parameter, i.e., the stress-coupling strength parameter β, was used to match the simulation data to the experimental results. Furthermore, in all fits shown in Fig. 9 (including the strong- and weak-constriction results) the stress-coupling parameter is the same, $\beta = 250$.

In construction of our model our goal was to make it realistic but as simple as possible, with a minimal number of free parameters.

To clarify the issue of the model parameters and the fitting procedure, a paragraph has been added to our discussion of the model to highlight that fitting was accomplished using a single parameter β. We also added a subsection at the end of the **Methods** section, in which we discuss the dimensionless parameters of the model.

2 (a) Reviewer's Comment: *Also for figure 9: In the fitting, is the model including feedback fitted to the data, and feedback then turned off to produce the curve without feedback, or are both scenarios somehow fitted to the data individually? I'd be worried that the former might introduce bias to the model comparison.*

Our Reply:
The feedback strength β is the only fitting parameter used in our model, as explained in Item 1 and in the new subsection of the **Methods** section. In effect, fitting is accomplished by varying how sensitive cells are to mechanical feedback. To produce the curve for random constrictions for a given value of the constriction strength f_c the feedback is turned off by setting $\beta = 0$ and keeping f_c constant.

We would like to reemphasize that all parameters of the model except f_c and β have the same values in all our simulations. They are based on considerations such as approximating the embryo geometry and are not used for fitting. Thus, the model without feedback is, essentially, parameter-free, and there is no bias because there is no fitting.

2 (b) Reviewer's Comment: *In your model, chains form always in antero–posterior direction. Is this simply a result of the imposed boundary conditions, i.e. the symmetry of the domain, or is there another, more explicit bias to introduce this anisotropy?*

Our Reply:
In our model, the anisotropy of the chains results exclusively from the geometry of the system: the boundary conditions and the presence of the passive region with no constrictions in the lateral domain. The boundary condition in the longitudinal direction mimics the interaction with the immobile end caps. The boundary condition in the transverse direction mimics the interaction of the active region with mobile elastic tissue of the embryo's lateral regions. Thus, the major stress and the precursor stress chains are primarily oriented in the anteroposterior direction, which results in the same predominant orientation of the constriction chains.

We have clarified this issue in the manuscript by adding a new paragraph touching on the anteroposterior orientation of the chains to the subsection which discusses the predictions of our AGF model.

3 (a) *Reviewer's Comment:* *Figure 3: After having stared at this for a while, I believe I can see the similarities you mention in the text, but then maybe I am only imagining things because I have looked at it for so long. Is there any way to make this more explicit? Maybe similarities can be highlighted with arrows or some such? Or perhaps it's possible to quantify the similarities as the proportion of cells at each time point that have already been counted as constricted at a previous timepoint, under a weaker cutoff? Also, I don't understand why the top right panels are not displayed.*

Our Reply:
We have modified the figure by adjusting the color scheme to help identify chains that persist across different cutoff values r_c. The top right panels are not displayed because at later times there are so many cells identified as constricting for the weaker r_c values that it becomes difficult to visually observe persistent CCCs.

3 (b) *Reviewer's Comment:* *On page 9, it is not clear to me why the potential between cells is defined as a piecewise function, since both pieces seem to have the same functional form. Is it so to clarify that only the attractive part of the potential is restricted to interactions with nearest neighbours? Since the range for the repulsive contribution is smaller than the range of the attractive contribution, won't the repulsive contribution only act on nearest neighbours anyway?*

Our Reply:
We have rewritten the description of the interparticle potential to make it more clear. Adjacent cells connected by adherens junctions are modeled using a spring potential with both the repulsive and attractive parts. Cells that are not adjacent interact only via the repulsive potential.

The non-adjacent cells are usually separated by a distance larger than the interaction range, so the repulsive potential does not affect their behavior. However, in other problems to which our model may be applied, non-adjacent cells may be able to interact when they change their positions due to relative particle motion. Moreover, the repulsive potential acting between all cells is needed during preparation of the initial state (i.e., before the adhesive forces are switched on). Therefore, we prefer to keep this potential to preserve our model's generality.

4. *Reviewer's Comment:* *Page 9, line 163: 'The normalisation by N_A ensures that approximately the same number of cells constrict in each time step.' Why is this important, is this the case biologically? Does the propensity to constrict increase for each cell as the process progresses in the real tissue? Can you comment on possible mechanisms for this?*

Our Reply:
To answer this question we first note that our simulations can be considered as a discretization of a continuous stochastic process in which constrictions occur instantaneously with a prescribed (stress dependent) probability per unit time. Thus we choose a normalization where only a small number of cells constrict in each simulation step (typically one or two). In this limit the normalization by N_a does not affect the system dynamics, provided that the results are presented vs the number of already constricted particles (rather than time or the number of simulation steps).

The normalization by the current number of active particles (rather than the initial one) is introduced for numerical efficiency: it allows us to avoid performing many numerical steps

with no constrictions at the late stages of the evolution, where there are not many active particles left. Since the normalization used does not affect the simulation results, it does not correspond to increased propensity of cells to constrict and therefore does not have any biological meaning.

The text below equation (8) has been revised to clarify this issue.

5. Reviewer's Comment: *Page 3, line 42 "…that these data are inconsistent with results for purely random uncorrelated constrictions, or for only neighbour-correlated constrictions" – Which part of the paper do you refer to with 'only neighbour-correlated constrictions'? This was not clear to me.*

   Our Reply:
   We refer to the supplemental figure S1 Fig, which shows that constricted cells form separate clusters rather than chains. Clarification of this has been added at the end of the paragraph discussing Fig. 5.

6. Reviewer's Comment: *Page 6, line 116 "…a typical vertex model likely would be inadequate for description of this system, whereas a particle-based model that treats cells as undivided entities described by their effective properties is more appropriate" – I agree that a vertex model would introduce complexity that is not necessary for your model, and that the particle based approach is better suited here. I don't think a vertex model would be inadequate though, I am quite convinced that a vertex model could well reproduce your results! Maybe rephrase this? Also, sorry for this comment, I use vertex models regularly, so I am obviously biased.*

   Our Reply:
   We agree with this comment. We have rephrased the paragraph in question accordingly.

7. Reviewer's Comment: *Is it possible to add colorbars to the figures where saturation denotes stress magnitude, such as in figure 8?*

   Our Reply:
   Colorbars have been added to the figures where saturation denotes stress magnitude.

8. Reviewer's Comment: *In Figure 13, can you indicate constriction chains in some way? It is difficult for me to see where constriction chains are in these images.*

   Our Reply:
   Since this figure is reprinted from a published work by Guglielmi et al. (2015), we prefer to leave Fig. 13 unmodified. However, we have added supplemental figures S4 Fig and S5 Fig in which constriction chains are highlighted.

9. Reviewer's Comment: *Will your simulation code be available somewhere? (I believe that is journal policy for PLOS Comp Biol)*

   Our Reply:
   Yes, it has been posted on GitHub.com and is now available. [Link]

Response to Reviewer 2

Response to general remarks

Reviewer's Comment: *This work by Holcomb and colleagues addresses the formation of the ventral furrow that occurs during fruit fly embryogenesis. The paper is a follow-up of Gao et al. 2016, from the same group. The authors of the cited paper proposed, based on computational experiments, that mechanical stress may have an instructive role in cell constriction and promote the formation of cellular constriction chains (CCCs) that are necessary for successful invagination of the embryo. Holcomb et al propose a quantitative comparison between the predicted dynamics and experimental observations, as well as new predictions on the robustness of the process.*

In the first part of the manuscript, they show the progressive formation of CCCs in the fly embryo using in vivo imaging and image analysis. Then they introduce a particle-based cellular model (based on Gao et al., 2016) allowing to model mutual cell repulsion and adhesion, as well as stochastic cell constriction as a response to mechanical stress. They derive a number of very interesting results. First, they propose that cells initiate CCCs according to an initial heterogeneous pattern of stress. Cells that are subject to higher tensile stress are more likely to shrink, which in turns amplify the nonuniformity of the stress field, in a potentially very general feedback loop. The authors also assess the robustness of CCC formation with respect to localised constriction defects, and demonstrate that a stress feedback allows to rescue normal cell constriction in a number of scenarios. This last result is compared to previously-published experimental analyses.

I found this paper very stimulating and interesting in general. The general mechanism proposed and explored by the authors is remarkably parsimonious, and seems in good agreement with experimental observations. I believe it would well fit the scope of PLOS Computational Biology. Nevertheless, I do have some concerns listed below, that would require major revision.

Our Reply:
We are glad that the Reviewer finds our paper stimulating and interesting and that he/she believes that the paper would fit the scope of PLOS Computational Biology. Our responses to the Referee's concerns are listed below.

Response to Reviewer's concerns

1 (a) Reviewer's Comment: *My major comment concerns the mathematical details of the modelling framework. I generally think that the modelling choices and the mathematical expressions chosen by the authors would deserve further justification.*

The distinction between Eq. 4a (repulsion) and 4b (attraction) is not clear to me, and it seems like a rather ad hoc choice. In particular, shouldn't we expect that $V_r(r_{ij})$ will be effectively zero if cells are not adjacent? In this case, combining the two energies into a single one:

$$V_{ij} = \frac{\epsilon}{2}\left(1 - \frac{r_{ij}}{d_{ij}}\right)^2$$

would probably be more straightforward. Also, why should adhesion only exist between adjacent cells and not repulsion?

Our Reply:
We have rewritten the description of the interparticle potential to make it more clear. Ad-

jacent cells connected by adherens junctions are modeled using a spring potential with both the repulsive and attractive parts. Cells that are not adjacent interact only via the repulsive potential. The non-adjacent cells are usually separated by a distance larger than the interaction range, so the repulsive potential does not affect their behavior. However, in other problems to which our model may be applied, non-adjacent cells may be able to interact when they change their positions due to relative particle motion. Moreover, the repulsive potential acting between all cells is needed during preparation of the initial state (i.e., before the adhesive forces are switched on). Therefore, we prefer to keep this potential to preserve our model's generality.

1 (b) Reviewer's Comment: *Why is the threshold chosen to be the mean diameter?*

For solid particles the particle radii are pair-additive. The mean diameter corresponds to touching undeformed particles. For smaller distances particles have to deform and therefore repel each other due to elasticity. For larger distances the particles do not touch and therefore do not interact.

1 (c) Reviewer's Comment: *As for the stress-based feedback: Why defining an additional triggering stress σ_i instead of taking the trace of the virial stress Si for instance?*

Our Reply:
We have used the triggering stress to maintain compatibility with our previous paper. The triggering stress differs from the trace of the virial stress only by normalization factors under the summation. There is no biological reason to choose one definition over the other.

2. Reviewer's Comment: *Eq. 9: Will $P_i(s_i)$ be always be ≤ 1?*

Our Reply:
Yes, for realistic stresses the probability will always be much smaller than 1. We have rewritten the paragraph below Eq. (8) to clarify this issue.

3. Reviewer's Comment: *The manuscript does not provide sufficient details on the choice of the simulation parameters or the numerical resolution. This is a limitation in so far as the authors claim to have a quantitative approach.*

For example, L197: I am surprised by the precision given for parameter $s_0 = 2.1$. Does it have a strong importance on the result?

In particular Fig. 9 presents quantitative results that show a good agreement between simulation and experiment. However, this result is predicated upon many parameters that are not clearly discussed.

This is a major limitation that I would like the authors do address.

Our Reply:
We have added a new subsection in the **Methods** section to discuss the dimensionless parameters of our model and the fitting procedure. In short, the fitting is performed using a single stress-coupling parameter β, and a single value $\beta = 250$ is used for all plots in Fig. 9. The parameter f_c by which the radius of the interaction potential is reduced upon constriction is determined by the strength of constrictions considered. The remaining parameters are either determined by the geometry of the system or are of technical nature and do not have a significant influence on our results. All these parameters are kept constant in our simulations and were not used to fit the data.

The resolution of our simulation data is marked by error bars indicating the statistical errors. The error bars in Fig. 9 are smaller than the size of the symbols showing the simulation data. This issue is clarified in the caption of Fig. 9 of the revised manuscript. Numerical inaccuracies associated with our energy minimization procedure are insignificant.

The parameter s_0 in the Voronoi tessellation procedure does not affect the quantitative results presented in Figs. 9, 10, and 12, because the constriction process and the connected neighbor list are independent of the Voronoi tessellation. The cell shapes in the simulation snapshots depend on s_0, but this dependence is not significant as long as we use $s_0 \approx 2$. We have clarified these issues in the added subsection in the **Methods** section. The specific value $s_0 = 2.1$ used in our simulations stems from a somewhat different normalization of virial stresses used in our numerical procedures. This exact value is inconsequential, and it has been de-emphasized in the revised manuscript.

4. Reviewer's Comment: *Please provide detailed information on the computational framework. Is the code available somewhere?*

   Our Reply:
   Yes, it has been posted on GitHub.com and is now available. [Link]

5. Reviewer's Comment: *L723: The rationale behind the choice a periodic boundary condition for the x-axis is not clear to me. Why not simply fixing the x-position of the boundaries? What kind of implementation diffculty would this bring?*

   Our Reply:
   Since the immobile end caps are in high curvature areas of the embryo, fixing the x-position on the boundaries of the rectangular simulation domains would not make the model more realistic. To make the system geometrically more accurate, the modeling would have to be done on a curved surface, and additional mechanical elements (such as the energy associated with curvature change) would need to be added to the model, making the system much more complicated. Moreover, the cells of the end caps still exhibit some mobility in the sense that they can be stretched as they are pulled by the constriction of cells in the active region.

   The periodic boundary was selected over a fixed one because we felt that it better approximates the conditions *in vivo*. The periodic boundary conditions serve well the purpose of creating conditions for propagation of tensile stress in the anteroposterior direction, and therefore we prefer to stay with the current modeling approach.

6. Reviewer's Comment: *L116: this statement is a bit weak. Constriction effects could in principle be incorporated into a vertex model, e.g. via an energy penalty on cell areas. I would suggest the authors to elaborate on this point. Also, further details on existing particle-based models would be useful.*

   Our Reply:
   We have revisited and rewritten this sentence. We believe that vertex models would be capable of representing a constriction process with mechanical feedback; they would, however, introduce additional complexity that can be avoided with our particle-based approach. A review article [Comp. Part. Mech. (2015) 2:401–444] describing existing particle-based models has been added to our reference list.

7. *Reviewer's Comment:* *Eq. 11, a reference on the notion of "virial stress" would be useful.*

   Our Reply:
   We have added an appropriate reference.

8. *Reviewer's Comment:* *Paragraph Optogenetic experiments provide evidence of robustness of apical constrictions in live embryos. The authors make several observations that should be better argued. I surely appreciate that quantification may be difficult. However, statements like "The lowest level of the optogenetic activation [...] reveal constriction chains crossing the affected region of reduced cell contractility in the middle part of the frame" or "the degree of cell contractility reduction is commensurate with the beam power" are not self-evident to me, based on the image provided.*

   Our Reply:
   In response to this comment we have added supplemental figures S4 Fig and S5 Fig in which we show a blowup of panels F,G,J,K from Fig. 13, with constricted cells manually indicated. We hope that these new figures make our interpretation of the optogenetic experiments clearer.

9. *Reviewer's Comment:* *Fig 13. Would a control case be available?*

   Our Reply:
   The control is, in fact, I-L in Fig 13 (from Guglielmi et al. 2015) because the 0.7 mW experiment is similar to the control "albeit with lower levels of a-p anisotropy when compared to WT embryos (Figures 5I-5L and 5O; Movie S5C)." (from Guglielmi et al. 2015). Fig 3 of Guglielmi et al. 2015 shows the wild-type control embryos for this experiment: embryos expressing both CRY2-ORCL and CIBN::pmGFP without illumination at 488 nm.

   The reference for a control case has been added to the **Results** section.

10. *Reviewer's Comment:* *L571–578: I do not understand this paragraph.*

    Our Reply:
    We have rewritten the paragraph.

11. *Reviewer's Comment:* *An element of discussion: could pulsatile constriction be a way to channel the formation of CCCs and improve the robustness of the process (by introducing some form of 'thermal' noise)?*

    Our Reply:
    Absolutely. We are working on introducing pulsatile constrictions to our model and believe it is likely that pulses are important for the readout of stress.

12. *Reviewer's Comment:* *L186: typo "the the".*

    Our Reply:
    The typo has been corrected.

13. *Reviewer's Comment:* *Fig. 1: Note that the bar-circle representation does not make it possible to really visualise the cell surface areas, which anyway do not provide much information I think.*

Our Reply:
This is correct. We felt that the visualization of the orientation of the major axis of the cell's shape was more informative than the visualization of the cell surface area. The blow up (orange box) provides an example of what the cell areas look like; however, our focus is on what we can learn from the anisotropy of the cell shapes rather than the areas themselves.

14. *Reviewer's Comment:* *Does ν_{avg} actually refer to $\%N_c$?*

Our Reply:
No, ν_{avg} is similar in spirit to $\%N_c$; however, it is based on information from a single frame (or subregion of a single frame) rather than from across the multiple frames of a time lapse.

The fraction of constricted cells ν_{avg} is defined as the number of constricted cells divided by the total number of cells in a single frame (or subregion of a single frame) while $\%N_c$ is the fraction of *active* cells that constricted. The active cells were determined by observing cell constriction activity across the entire time lapse sequence of images. The frames where ν_{avg} was implemented were stand-alone images which meant that there was no way to establish the number of active cells.

To clarify this we have revised our introduction of ν_{avg} and specified that it differs from $\%N_c$.

Response to Reviewer 3

Response to general remarks

Reviewer's Comment: *The spatio-temporal organization of stress and deformation during a Drosophila embryo's ventral furrow formation is an important topic in current developmental biology. The manuscript by Holcomb et al. centers on the application of theoretical models from the physics of granular fluids to identify the role of mechanical regulation of cellular behavior – namely, the onset of apical constriction. In previous work, the authors developed a computational model of the ventral furrow region in which the chain-like organization of constricting cells (as seen in experiments) occurred when local stress directly promoted contraction. In the current manuscript, this model is used to make a number of quantitative predictions which are directly verified with experimental data. The computational model is shown to successfully predict the size distribution of constricting clusters within the VF region, and further the authors demonstrate that stress-based feedback promotes cluster formation in the presences of local contractility perturbations. While I have some specific comments and suggestions, I overall recommend this paper for publication.*

Our Reply:
We thank the Reviewer for these positive comments.

Response to comments and questions

1. Reviewer's Comment: *The authors note that they choose to use their AGF model instead of a more commonly used vertex model. I think the model they use is reasonable, especially in light of the experimental data, but I would like to see a bit of expansion of the paragraph addressing the differences between said models. In particular, I do not entirely understand the claim: "A typical vertex model likely would be inadequate for description of this system, whereas a particle-based model that treats cells as undivided entities described by their effective properties is more appropriate." Is there a reason to think that a vertex model under similar boundary conditions, and with similar stress-based feedback, would not exhibit contractile chains?*

   Our Reply:
   We have revisited and rewritten this sentence. We believe that vertex models would be capable of representing a constriction process with mechanical feedback; they would, however, introduce additional complexity that can be avoided with our particle-based approach.

2. Reviewer's Comment: *The stress-modified Voronoi tessellation the authors develop make intuitive sense to me, given significant role of stress in determining the shape of cells in vivo. However, I believe it would strengthen the paper to introduce a quantitative basis for the statement "We find that the augmented Voronoi algorithm renders a realistic representation of a confluent cellular medium with a significant degree of polydispersity and local anisotropy associated with the presence of CCCs". For instance, if you take the cell positions from Fig. 2 and assuming the stress can be calculated from these positions using the AGF force terms, how much deviation is there between the Voronoi calculated cell shapes and real ones? I also think a cartoon illustrating the tessellation calculation might be helpful if added to Fig. 4.*

   Our Reply:
   Our model cannot be used to determine stresses in live tissue, because we do not have

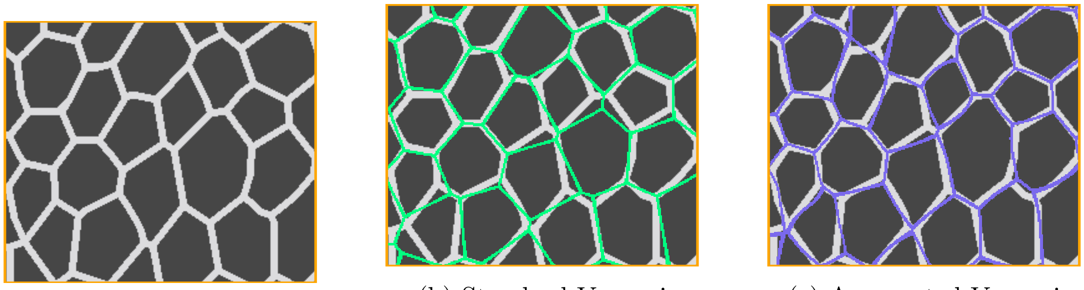

(a) Segmented embryo image without tessellation.

(b) Standard Voronoi tessellation overlaid over the embryo image.

(c) Augmented Voronoi tessellation overlaid over the embryo image.

FIG. 1-R3: Voronoi reconstruction of cell shapes *in vivo* shown in panel (a) from the cell centers (b) and the cell centers and second-moment shape matrix (c).

information about equilibrium cell sizes in the absence of inter-cellular forces (the model was not designed for this purpose). In principle, cell shapes can be calculated from cell positions using the second-moment matrix of the cell shape as the input to the Voronoi algorithm. A result of such reconstructions is depicted in Fig. 1-R3 of this response. However, such analysis is beyond the scope of the present study.

To follow the Reviewer's recommendation and to show the effect of the stress contribution on the Voronoi tessellation, we have thus used a different approach: in the new supplemental figure S2 Fig we compare the standard Voronoi tessellation, polydisperse tessellation (which takes into account different radii of the interaction potentials d_i), and our new stress-based tessellation. Only the stress-based tessellation predicts realistic elongated shapes of constricted particles. We hope that the Reviewer will find this figure sufficiently informative.

Following the Reviewer's recommendation we have added to Fig. 4 a schematic explaining the stress-based Voronoi tessellation.

3. Reviewer's Comment: *It seems like particles keep the same neighbors over the course of each simulation, but it would be good to clarify this point if true. The lack of T1 transitions and other cell rearrangement is an important feature of tissue dynamics at this state, so if this is not being enforced it should at least be commented on.*

   Our Reply:
   The epithelial structure of the blastoderm is maintained throughout VFF. To reflect this feature in our model, we establish the connected neighbor list from the initial configuration and maintain it without change throughout the entire simulation. We state in the caption for Fig. 4F that cells maintain the same neighbors even after constriction. We have also added a paragraph explaining this feature to the **Model** section of the revised manuscript in the interest of clarity.

4. Reviewer's Comment: *Fig. 9 is reasonably convincing evidence, but it would be useful to have additional metrics for comparison between experiment and simulation, either in the main text or in the SI. For instance, it would be helpful to see a direct quantitative comparison at this point of the AP directional bias of the clusters for the cases presented in Fig 9.*

Our Reply:
We thank the Reviewer for this suggestion. Our analysis of the directional bias is presented in the revised manuscript in the supplemental S1 Appendix.

5. Reviewer's Comment: *Visually, it would be good to include a horizontal dotted line denoting a_r in Fig. 10 C.*

   Our Reply:
   The figure has been updated to include lines denoting the a_r.

6. Reviewer's Comment: *Generally, attempts to demonstrate robustness involve demonstrating successful mechanical behavior in the context of plausible perturbations to the in vivo system. The discussion of robustness is mainly carried out in relation to an optogenetically controlled mutant embryo, which represents a more contrived sort of perturbation than a simple genetic change. As I read it, the authors argue that their experimental data should be taken as representative of a general class of mutations which undermines contractility in a local region of cells. To better shore up this point, it might be good for the authors to include references to other mutants which exhibit regions of low expression in the ventral furrow region.*

   Our Reply:
   We disagree with the Reviewer. Plausible perturbations include more than simple genetic changes. Stochastic biochemical and genetic abnormalities can produce perturbations that are compensated for by robustness in the system. Other environmentally caused perturbations can include temperature, pH, chemicals, toxins, etc. In its environment, the embryo may be subjected to mechanical perturbations that robustness counters. Many studies of mechanical perturbations have been conducted over the past century. We consider the optogenetic experiments of Guglielmi et al. (2015) to be an elegant and tunable perturbation that can simulate a variety of biochemical, genetic, mechanical, and other environmental problems. The Guglielmi et al. (2015) system can be modulated in time, intensity, and developmental stage, in either specific parts of the embryo or the whole embryo. With respect to the Reviewer's comments regarding the optogenetic data being taken as representative of mutations that affect contractility in a specific region, we note that various mutations in sna and twi meet this criterion, as well as several members of the dorsoventral patterning genes. Furthermore, the reduction of activity of actin, myosin, actomyosin regulators such as Rho or Rok, or of adherens junction components such as Shg (E-cad) or Arm could be modeled by the optogenetic approach. We have included a comment on this in the **Discussion** section.

7. Reviewer's Comment: *The modification of the model in the robustness section reduces the contraction probability of particles, but intuitively I would expect the reduction of myosin recruitment observed in the would also influence the magnitude of the contractions observed in constricting cells. The authors should comment on this, as I would assume that if the computational model incorporated reduced contraction in the perturbed region, the corresponding stress and thus stress feedback effect in this region would likewise be diminished.*

Our Reply:
Our model predicts that the stresses in the affected region originate from constrictions in the unaffected domains due to long-range stress propagation. This phenomenon is highlighted in the first frame of Fig. 11. Only after sufficiently strong stresses build up along the stress chains in the affected region, do cells in this region also start to constrict, rescuing the constriction process and enhancing the tensile stress.

As seen in Fig. 13 [the image reprinted from Guglielmi et al. (2015)], cells in the affected region can constrict strongly (panel G), but such constrictions occur with a delay necessary for stress buildup. For example, compare the number of constrictions between F and J (constrictions marked in S4 and S5). The affected cells can even hyperconstrict (panel H) at times that are much longer than the usual invagination time in wild-type embryos. Therefore, in our model we reduce the constriction probability but not the degree of constrictions. We have clarified this issue in the manuscript.

Please note that our model is general, i.e., it is not meant to represent any specific mechanism of the reduction of cell contractility. It illustrates the principle: stress chains generated by constrictions of unaffected cells penetrate the affected region and rescue the constriction process by enhancing the contractility of the affected cells.

Response to Reviewer 4

Response to general remarks

Reviewer's Comment: *Holcomb et al present a largely model-based paper, but with some image quantification from published work, arguing that cell constriction during ventral furrow formation in Drosophila involves mechanical feedback, resulting in chains of constricted/ing cells.*

Our Reply:
We disagree with this assessment of the content of our work. It seems that the Reviewer has missed the fact that Figs. 1–3, 6, 7, and 9 show our original experimental data. We provide this information in the first paragraph of the **Results** section and in the **Experimental methods** section. Only the results of optogenetic experiments that are depicted in Figs. 13 and 14 were obtained from published work and images provided by Dr. De Renzis. Thus our paper has a significant experimental component in addition to the extensive modeling effort.

Reviewer's Comment: *This is a very interesting and topical area, so I was very interested to review this paper. It is clear, nicely presented and I found it easy to read, but in its current form I found it unconvincing.*

Our Reply:
We appreciate the Reviewer's positive remarks that the topic of our paper is interesting and the paper is nicely presented. We hope that our responses to his/her specific comments will convince the Reviewer that our work provides strong arguments for the presence of mechanical feedback in VFF and that the paper deserves publication in PLOS Computational Biology.

Response to Major Points

1. Reviewer's Comment: *A feature that worried me throughout the whole paper was that in vivo, cells nearest the ventral midline have the earliest and strongest apical Myosin activity (Xie & Martin) and start pulsatile and ratchetted constriction first. This activity subsequently spreads away from ventral towards the lateral edge of the mesoderm domain. So the effective width of the domain of contracting cells is rather narrower than the 12 cells the authors use in their simulations. From the in vivo data in Figs 1,2,3 & 7, the active domain that shows DV constriction over the developmental epoch presented is around 6 cells wide, and rather than showing separated chains of contracted cells, it is more like a block of cells, with many connections that are not along AP. This does not really agree with the patterns of simulated chains that emerge, for example, in Fig 8.*

   Our Reply:
   The width of the active region in our simulations was selected in line with the findings of Sweeton et al. (1991) who reported that the domain is actually 18 cells wide but flattening is initially only apparent in 12-15 cells. Since we focus on the initial slow phase of apical constrictions, a 12-cell-wide active region was used.

   In our analysis of embryo images we apply specific constriction criteria based on changes of the cell minor axis (rather than the cell area). These criteria yield a narrower field of active cells. For example, in the frames depicted in Fig. 2 we have detected approximately 140 active cells, which form a narrower band than the 12 cells assumed in our numerical model. However, since simulations with a narrower active region do not change our key quantitative

results shown in Fig. 9 (see Fig. 1-R4 below and Item 3 of this response), the conclusions presented in the manuscript are not affected by the choice of the active region width.

Our experimental images (Figs. 1,2,3 and 7) show formation of chains, as predicted by our model. However, at the end of the constriction process the chains tend to combine into a more compact structure due to a gradual transition to the fast phase of VFF, when isotropic Fog signaling becomes important. We agree that cell contractility is larger near the ventral midline, but our model does not consider this effect. Therefore, Fig. 8 shows chains that are more uniformly distributed.

The spatial variation of cell contractility and biochemical signaling can be readily incorporated in our model. However, these extensions would require us to bring in additional fitting parameters (e.g., to describe the degree of the constriction bias). Our current AGF approach was developed to obtain a simple model with a minimal number of free parameters. Hence we use only one free parameter β (the stress-coupling strength parameter) to fit multiple elements of the measured cluster size distribution (Fig. 9). If needed, our model can be refined in our future work; we indicate this in the last paragraph in the **Discussion** section.

2. Reviewer's Comment: *Some detail is shown in Figure 7 of chains supposedly emerging in vivo. The central chain was plausible but I could not understand how chains could branch off in DV-oriented directions when tension is supposed to be along AP.*

   Our Reply:
   The overall orientation of the tension is in the AP direction; however, the stress field is fluctuating. Such local fluctuations can result in branching of the stress chains. This kind of behavior is a well-known feature of particulate media; please see Figs. 1 and 2 of "Contact force measurements and stress-induced anisotropy in granular materials" by Majmudar and Behringer, Nature, 2005. [Link] Local fluctuations are also clearly visible in the stress distribution depicted in Figs. 10–12 of our manuscript.

3. Reviewer's Comment: *So, given that this is just an illustrative example, the proof should be in the summary of quantified in vivo and simulated data in Fig 9. Here, to be convinced of tension propagation, I would need to see results for simulations that focus only on the most ventral 6 rows of cells, using etas and %Nc for just this region, not including all the non-chaining lateral cells, which would mean a much more dense field of constricting cells. In each of the left hand graphs (A,C,E,G), I could see the pink star line (no mech feedback) moving towards the purple triangle line (mech feedback) in this scenario. So I am not yet convinced that the data support mechanical feedback.*

   Our Reply:
   In response to this comment we have run and averaged multiple iterations of the simulation with the width of the active region reduced to 6 cells; the results are presented in Fig. 1-R4 below. The plots of our new results are overlaid on panels from Fig. 9 for reference. The data show that the reduction of the width of the active region does not cause the random constriction line (i.e. no mechanical feedback) to collapse towards the tensile feedback line as suggested by the Reviewer. The concern expressed by the Reviewer is thus not valid.

4. Reviewer's Comment: *The right hand graphs (for weaker constrictions) clearly do not favour mech feedback. I think it's also true to say that the evidence in Xie and Martin (2016) for chains of constricting cells is quite weak, and that the orientation of chains that they show are*

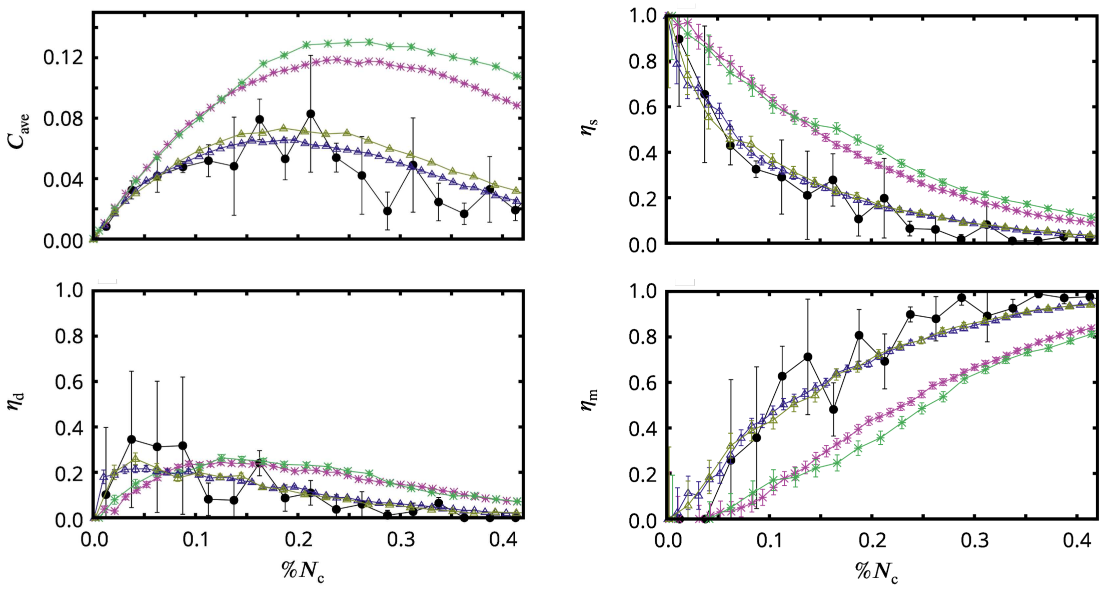

FIG. 1-R4: **Constricted-cell cluster counts for a system a with six-particle-wide active region.** The experimental and simulation data are for the strong-constriction case. The results are overlaid on the panels A,C,E,G from Fig. 9 for reference. Black circles represent the experimental results. Purple and yellow triangles represent tensile feedback simulations with 12-cell-wide and 6-cell-wide active regions, respectively. Pink and green stars represent random constrictions with 12-cell-wide and 6-cell-wide active regions, respectively. All model parameters (except for the width of the active region) are the same in the 12-cell-wide and 6-cell-wide systems.

not strongly biased to be AP-oriented. So I would downgrade this evidence to being suggestive of the possibility of mechanical feedback, but biochemical communication (via extracellular fog or via more direct cell-cell signalling) or entrainment of biochemical oscillators or . . . are also possible explanations.

Our Reply:

For strong constrictions the difference between the random and mechanical-feedback curves is much larger than for the weak constrictions, because stresses generated by the cells are larger and therefore provide stronger feedback. The experimental data show exactly this expected trend, consistent with our model. The reason that we show cluster counts for both strong and weak constrictions is that taken together they strongly support our conclusions.

Regarding the Reviewer's remark about the results of Xie and Martin (2015), we note that these authors did not look for chaining behavior. In particular, they did not examine any multiparticle correlations or shapes of constricted particle clusters, in contrast to our study. Instead, they focused on pairwise correlations between contractile pulses of neighboring cells. Also, they did not present any angular distribution data. Moreover, their field of view was too small to observe chaining. Their results thus do not contradict or weaken our conclusion. The data and simulations we present here allow us to observe chaining, because we analyzed a large field of cells.

Xie and Martin (2015) argue for a mechanical coordination of neighboring cell constrictions: "The results are consistent with a 'neighbor-activating' model of collective cell shape change,

where a central cell's ratcheted constriction pulse could promote contractile events in neighbors, either through mechanical tension or cell shape strain." They cite several sources in support of this mechanism. This statement strongly suggests that had the Martin group obtained data similar to ours, they would support a tension feedback mechanism.

Extracellular Fog plays a major role in the fast phase of VFF, but we see no need to invoke smaller quantities of Fog inducing pulsatile ratcheted constrictions in the slow phase of apical constrictions in VFF. This would complicate our model but would not change our conclusions. Direct cell–cell triggering would not produce percolating chains, as evident from S1 Fig of our study. The biochemical mechanisms mentioned by the Reviewer are unlikely to induce chaining of cellular constrictions and to explain the results of optogenetic experiments by Guglielmi et al. (2015), where the mechanical force transmission was directly affected.

5. Reviewer's Comment: *Surely if tension chains were real and as clear as the authors suggest, that paper would have found much stronger evidence?*

   Our Reply:
   Our experimental data presented in Fig. 2 clearly demonstrate the development of CCCs. Furthermore, our experimental results in Fig. 7 show that CCCs are not formed by apically constricting cells causing the apical constrictions of their neighbors but instead by both near and distant apically constricted cells that gradually link up into chains. This phenomenon reveals the role of tensile stress that can correlate apical constrictions at a distance (Fig. 8).

   We believe that our arguments showing the formation of constriction chains induced by mechanical feedback are strong. Using a single value of the only fitting parameter we can describe multiple data sets, and many qualitative features of the observed constriction patterns are reproduced by our model. Moreover, our simulations show that the chains form in a system with tensile-stress feedback (Fig. 5A) but not in a system with random constrictions (Fig. 5B), nor as a result of neighbor-triggered constrictions (S1 Fig).

   As noted in Item 4, the fact that Xie and Martin (2015) did not observe chains does not weaken our arguments. They analyzed only pairwise correlations between contractile pulses in neighboring cells, a valid approach that enabled a better understanding of apical constrictions. In their experiments, they only looked at a cell and its neighbors so as to be able to follow the ratcheted apical constrictions at high resolution. However, this approach precluded the ability to look at a larger field of cells. Had Xie and Martin looked at the whole field of the ventral primordium, they likely would have observed the apical constriction chaining phenomenon and likely attributed it to tensile stress.

6. Reviewer's Comment: *It's possible of course that there is some weak tension propagation, but I think the tools you would need to disentangle this would be local mechanical perturbation in vivo, cutting across single 'chains' with laser ablation for example.*

   Our Reply:
   We contend that the optogenetic data of Guglielmi et al. (2015) show strong evidence for this tension propagation. By locally disabling the actomyosin contractility in specific cells, they created local mechanical perturbations *in vivo* that do cut across chains. As reported by Broadland et al. (2010) in their presentation of video force microscopy, relative changes in cell shape through time-lapse images reveal information about the forces that drive those changes. In a similar spirit, the variance in how cellular shapes develop between different

levels of optogenetic perturbation (both within and outside of the photoactivated areas) provides strong evidence of tension propagation through the domain.

We agree with the Reviewer that further experiments *in vivo*, such as those proposed in his/her comment, are needed to provide additional insights into stress propagation in the tissue and its role in development of the constriction patterns. We indicate the need for such experiments in the **Discussion** section of the revised manuscript. We note that our current results will help design such experiments (which are, however, beyond the scope of the present study).

7. Reviewer's Comment: *The authors seem to be claiming that in vivo cell contractility (in DV typically) is a pure readout of stress, whereas surely the effective material stiffness can also vary, and locally? What would the implications be for their model and for the interpretation of cell shapes of locally varying effective cell stiffness?*

   Our Reply:
   We are not aware of any evidence that the effective material stiffness has a significant effect on constriction patterns and we do not expect that stiffness variation would produce constriction correlations described in our work. Therefore, we do not see any reason to introduce novel regions of varying stiffness into the model. If needed, material stiffness variation can be added by changing the spring constant values. This modification, however, would only increase the complexity of our model, and we prefer to keep it as simple as possible, with a minimal number of free parameters.

8. Reviewer's Comment: *I would have thought that contractility (negative strain) by some cells in DV would help other nearby cells also contract in DV (cell adhesion is strong and cells don't slide, as the authors note) in order to maintain tissue integrity.*

   Our Reply:
   As seen in Fig. 6 (both in simulations and *in vivo*), membranes common to the constricted and unconstricted cells are reduced in length, but the overall width of unconstricted cells is not reduced. Constrictions of adjacent cells are thus not required for tissue integrity. Therefore, our results do not support the Reviewer's hypothesis about contractility interactions of nearby cells.

9. Reviewer's Comment: *When it came to the later sections of the paper on the function of tensile feedback, I'm not sure I yet buy the premise nor agree with the evidence. From the Martin et al. (2009) paper, apical constriction of the most ventral cells happens remarkably quickly, over around 6 ratcheted pulsatile contractions. I'm not sure this is long enough to really benefit from repair through tensile feedback, and the more relevant feature that ensures robust morphogenesis is the spatiotemporal gradient of actomyosin activation from ventral-most cells first through to more lateral cells later.*

   Our Reply:

   Our imaging data show that constriction chain formation occurs during a period of several minutes (Fig. 2). Thus, the chain formation time is similar to the time of ratcheted pulsatile constrictions reported by Martin et al. (2009). Importantly, one can also estimate that stress

propagation in the tissue is much quicker, as it occurs on the timescale of a fraction of a second, much faster that ratcheted apical constrictions.

There exists significant evidence that cells can react to their local environment on the timescale of ratcheted constrictions. For example, the correlations of constrictions of neighboring cells develop within the above time according to the results of Xie and Martin (2015). Since the timeframe of CCC formation is the same as that of ratcheted pulsatile constrictions, cells in a region of reduced contractility have sufficient time to react to the rapidly propagating mechanical signals associated with cell constrictions. This allows the constriction chains to penetrate or bypass the affected region, as demonstrated by our analyses of the results by Guglielmi et al. 2015 (Ref. 25) presented in Fig. 13.

We would like to emphasize that robustness to a partially reduced contractility in a local region does not require any independent repair mechanism activated by tensile stress (such activation could indeed require a longer time). Instead, the affected cells invoke the already active constriction machinery more strongly in order to compensate for the contractility reduction, which is shown by the work of Guglielmi et al. 2015 (Ref. 25).

The actomyosin activity gradient studied by the Martin lab is important for function and robustness in VF invagination (Ref. 28). However, more recent work by the Martin lab (Ref. 25) indicates that robustness is provided by multiple paths of contractility along the AP axis. This result is consistent with our findings.

10. Reviewer's Comment: *Would it not be a worry that mechanically propagated permanent tensile chains would be a dangerous positively reinforcing runaway process? Though there is no consensus yet on the function of cell pulsatility (hence this submitted paper), but it may be there precisely to ensure that chains are ephemeral/short-lived, to ensure coordinated tissue contractility that does not "runaway".*

    Our Reply:
    We agree with the Reviewer that pulses are most likely important; however, our simulations do not support the Reviewer's hypothesis that tensile chains with permanent constrictions would lead to a dangerous runaway process. To the contrary, the chains aid robustness of VFF, because tensile feedback promotes formation of a percolating network of constricted cells and chain penetration through regions of reduced contractility.

11. Reviewer's Comment: *Because actomyosin contractility would be the means through which tensile chains would be set up, I found it difficult to imagine an in vivo scenario where actomyosin contractility was sufficiently impaired locally that it was knocked out, but then able to be rescued by mechanical induction.*

    Our Reply:
    Buffering/compensation does not need to work all the way to aid robustness of biological processes. Biological buffering also compensates small fluctuations (similar to buffering in chemistry). We have clarified this issue in the manuscript.

12. Reviewer's Comment: *Maybe the separate roles of Twist and Snail in the ratchet and generating fluctuations would be a candidate. Adam Martin's group have shown that they can start cell fluctuations through physical perturbation when they are absent, but that is not really equivalent to showing tensile feedback of the kind required for chains.*

Our Reply:
The Reviewer's speculation on the separate roles of Twi and Sna in generating fluctuations is well taken. However, we are not directly addressing the molecular mechanism in this paper. There are many possible molecular mechanisms that likely work together in varying degrees with physical forces to propagate tension. Besides the effect mentioned by the Reviewer, important mechanisms also include orientation of actomyosin filaments, contractility gradients, redundant supracellular actomyosin filaments, directional stiffening, Rho dynamics, F-actin turnover, adherens junction connections between cells, and the mechanosensitive adherens junction-F-actin complex. Importantly, many of the molecular elements listed form or act upon the structure of the VF cells and are therefore acted upon by mechanical forces [Refs 1-4, 6-10, 16, 25, 28, Jodoin et al. (2015), Vasquez et al. (2014)]. In light of all of these, we have decided not to speculate on molecular mechanisms in this paper, but instead to analyze the mechanical behaviors of the VF forming cells.

13. Reviewer's Comment: *For the Fig 13 de Renzis panels, I didn't really see any chains in the 0.7 mW panels J & K, nor did I understand how the authors explain, according to their tensile chains idea, how the normal tissue in Fig. 13H, either side of the mid-section, becomes quite perturbed, with some larger and some smaller cells like the mid-section. . . . The patchwork of contracted cells amongst unusually big cells does not seem to me to fit with the tensile chains hypothesis.*

    Our Reply:
    We disagree. Our contention is that the visual patchwork paired with the similarity between the affected and unaffected areas supports our hypothesis. The optogenetic disruption does not only change how the cells in the affected area behave, but it modifies the adjacent unaffected areas as well. The optogenetically affected cells in the 1.5 mW embryos (Fig. 13H) are disrupted enough to impair the formation of the underlying stress field without fully suppressing it. Cells in the unaffected region react to the lack of "pull back" from the affected region by hyperconstricting. This produces the visual patchwork of hyperconstricted and enlarged cells while simultaneously promoting a buildup of stress upon the affected region. Once enough stress is applied, cells begin to constrict along the underlying tensile-stress chains that have penetrated the affected region. Note that the optogenetically unaffected regions of Fig. 13 H also show that cells that have not constricted have become enlarged, just as seen in the affected regions.

    In the supplemental material (S4 Fig and S5 Fig) we have presented enlarged images of Fig. 13 F,G,J, and K with constricted cells marked. The images show the cellular constriction chains spanning the optogenetically affected zones in both the moderately affected sample (1.5mW) and the control sample (0.7 mW) by pointing out the constricted cells.

14. Reviewer's Comment: *Their quantification of the 'chains' in Fig 14A & B shows that it's a network, without any long oriented chains. Shouldn't there be a trough of rescued chains rather than a peak in the centre of the graph in Fig 14B (top panel), as seen in the more extreme bottom panel?*

    Our Reply:
    The trough in the strongly photoactivated region in the 3.0 mW embryo demonstrates the extreme disruption of the ability of the active cells to constrict. This disruption is sufficient to prevent constriction of cells in the affected region. The lack of a trough associated with

a deficit of constricted cells in the moderately photoactivated region in the 1.5 mW embryo (top panel) is indicative of significant constriction rescue. The observed mild increase of constrictions in the affected domain may result from random fluctuations (we have only three images available for analysis), from the wider field of view in the center of the image (which can introduce some inaccuracies of cell counts), or from the geometry of chains not exhibiting the same degree of AP bias due to the optogenetic perturbation.

15. Reviewer's Comment: *Overall, the authors still have quite a lot to do to convince that tensile stress chains are a feature of ventral furrow formation.*

    Our Reply:
    We hope that our responses to the Reviewer's concerns and the corresponding revisions in the manuscript have convinced the Reviewer that our paper is suitable for publication in PLOS Computational Biology.

Response to Minor Points

1. Reviewer's Comment: *Lines 158-161 are crucial for following the thrust of the paper and should be introduced much earlier.*

   Our Reply:
   A statement of this information has been added to the **Introduction** so that it is provided earlier.

2. Reviewer's Comment: *Line 207, I didn't understand 'polydispersity'*

   Our Reply:
   A clarifying comment has been added.

3. Reviewer's Comment: *I found the paper long-winded in sections covering Figs 10–14. Salient features could be brought out more succinctly.*

   Our Reply:
   Throughout the paper we strived to be both concise and informative. We believe that the details provided in the sections covering Figs. 10–14 are necessary for understanding the stress propagation, the associated feedback, and its effect on the robustness of the constriction process. We thus prefer to leave these sections unchanged.
