# Supplementary material for: Mechanical feedback and robustness of apical constrictions in Drosophila embryo ventral furrow formation
Source: PLoS Comput Biol. 2021 Jul 6;17(7):e1009173. doi: 10.1371/journal.pcbi.1009173 (PMC8284804; doi:10.1371/journal.pcbi.1009173)
Supplement: Attachment — Submitted filename: Responses_1-4.pdf [file pcbi.1009173.s011.pdf]

Response to Reviewer 1

Response to general remarks

Reviewer's Comment: The authors have addressed all my comments very clearly and thoroughly. As I described in my previous review, I think this is an excellent paper that would make a strong contribution to Plos Computational Biology. I hence recommend it for publication.

Our Response:
We thank the Reviewer for the positive comments and appreciate the recommendation for publication.

Response to specific comments

1. *One little thing I noticed that I would hope can be addressed without further revisions is the following: The authors now explain the parameterisation of the model and the fitting very clearly in the paper. However, there is a parameter called epsilon in cell-to-cell force potential that appears to have been left out of these explanations. In the revised paper, I could not find the value that was chosen for this parameter epsilon and why it was chosen. Perhaps the authors can add a simple one or two sentences about this parameter in the final publication. In any case, I would assume that this parameter only controls the characteristic timescales of the dynamics, not the geometrical effects analysed in the paper.*

   Our Response:
   As correctly noted by the Reviewer, the geometrical characteristics analyzed in the manuscript are independent of the dimensional factors: the average particle diameter d and characteristic energy scale ϵ. Our results are presented in a dimensionless form; hence, they depend only on dimensionless groups.

   For example, the stress feedback parameter s is defined as the ratio between the current stress σ_i and the reference stress σ_{ref} determined from a system with a single constricted particle. While both σ_i and σ_{ref} depend on the dimensional factors d and ϵ, in the stress-coupling parameter s these dimensional factors cancel out, and therefore they do not influence the constriction process.

   Since the magnitude of ϵ does not influence the results presented in the manuscript, we do not give its value. In the future this coefficient will provide a means of setting the scale of interparticle interactions in the model, so that predicted forces, stresses, and energy can be compared to those *in vivo*. However, there are currently no experimental measurements of such values for *Drosophila* embryos for us to use as a basis to determine the parameter ϵ.

   We have added a paragraph to the manuscript clarifying that the geometrical characteristics of the constriction process are independent of the dimensional factors d and ϵ.

Response to Reviewer 2

Response to general remarks

Reviewer's Comment: *I thank the authors for the substantial work they have provided, which improves a lot their manuscript.*

Our Response:
We thank the Reviewer for these comments.

Response to specific comments

1. *FIG 13: I maintain that the paper should be self-contained as far as possible and that the control from Guglielmi et al. should be added to the figure (or as a Supp fig).*

   Our Response:
   We agree with the Reviewer's comment and have added a supplemental figure with the representative control embryo from Guglielmi *et al.*

2. *L181: Mathematically this is an affine relation (not linear).*

   Our Response:
   We agree that the relation is more correctly described as affine and have removed the word linear from the identified sentence.

Response to Reviewer 3

Response to general remarks

Reviewer's Comment: The authors have thoroughly addressed my comments from the previous review, and I recommend this revised version for publication.

Our Response:
We thank the Reviewer for his/her recommendation for publication.

Response to Reviewer 4

Response to specific comments

1. *Reviewer's Comment:* *Points 1 & 3: relating to the 6 vs 12 cell wide domain. The central argument of the paper hinges on Figure 9 which the authors claim shows that their model with tensile feedback closely matches in vivo data. Prior to Figure 9, the comparisons between model and in vivo data are visual, but in Figure 9 the comparison becomes objectively quantitative. I raised the issue in my review that because constricting cells in vivo are mostly packed into a central 6 cell wide tranche of the tissue, rather than throughout the 12 cell wide domain of Twist the authors have replicated in their model, would this change the interpretation of Figure 9? I thank the authors for repeating their model for the 6 cell wide domain, but unless I have missed something, for comparison to data they should adjust %Nc for the in vivo data too (in Fig 1-R4). %Nc is the "fraction of active constricted cells", the number of constricted cells divided by the total number of cells in the active region. Since the latter denominator has halved in the 6 cell domain, the in vivo data should also be recalculated with the (approx.) halved denominator. This will have the effect of stretching the in vivo data to the right in the 3 eta panels in Fig 1-R4.*

   For the the first C(ave) panel, there is a double effect on th denominator of C(ave) as well as on the %Nc x-axis. Could the authors please make this adjustment to see how the 6 cell wide model compares to 6 cell wide in vivo data? This should then be incorporated into Figure 9 and the conclusions adjusted as necessary.

   Our Response:
   The normalization of the experimental results in Figs. 9 and 1-R4 is correct and provides a meaningful comparison between the numerical simulations (both with the 6 and 12 cell-wide active domains) and the experiments. The fraction of constricted active cells $\%N_{\mathrm{c}}$ and the normalized number of clusters C_{avg} do not need to be adjusted for the *in vivo* data by reducing the number of active cells N_a by half (the change suggested by the Reviewer), because N_a is directly measured for each embryo and does not depend on the assumed width of the active domain in the numerical model.

   To determine the number of active cells in a given embryo, we directly count all cells that exhibit contractile activity (ratcheted and non-ratcheted) during the entire time-lapse before the onset of the fast constriction phase. As described in the manuscript, we use the minor axis reduction by a factor of at least $r = 0.85$ as the constriction criterion. The fact that the width of the active region in the model is reduced from 12 to 6 cells has no bearing on what is measured from our live embryos. Artificially halving the number of active cells *in vivo* when calculating $\%N_{\mathrm{c}}$ and C_{avg} would therefore be incorrect; it would yield an inaccurate representation of our experimental results. Hence, there is no need to replot our data or to change our conclusions.

Response to general remarks

Reviewer's Comment: *If the conclusions of Fig 9 still hold as the authors currently have them, or if my reasoning above is awry, then I am happy with the authors' responses to my other points.*

Our Response:
We thank the Reviewer for his/her additional comments.
